# Trajectories of work ability and associated work unit characteristics from pre-COVID to post-COVID pandemic period

**DOI:** 10.1136/oemed-2024-109475

**Published:** 2024-11-13

**Authors:** Johanna Kausto, Jaakko Airaksinen, Tuula Oksanen, Jussi Vahtera, Mika Kivimaki, Jenni M Ervasti

**Affiliations:** 1 Finnish Institute of Occupational Health, Helsinki, Finland; 2 School of Medicine, Institute of Public Health and Clinical Nutrition, University of Eastern Finland, Joensuu, Finland; 3 Department of Public Health, University of Turku, Turku, Finland; 4 Centre for Population Health Reserach, University of Turku, Turku, Finland; 5 University of Helsinki Clinicum Unit, Helsinki, Finland; 6 Brain Sciences, University College London, London, UK

**Keywords:** Public health, Epidemiology, Occupational Health, Statistics

## Abstract

**Objectives:**

To identify trajectories of work ability from pre-COVID to post-COVID-19 pandemic period and to examine work unit characteristics associated with these trajectories.

**Methods:**

The study population was a cohort of Finnish public sector employees (n=54 651) followed from 2016 until 2022. We used trajectory analysis to identify trajectories of work ability and multinomial regression to examine their associations with prepandemic work unit characteristics and pandemic-related changes at workplaces.

**Results:**

We identified three trajectories of work ability: (1) suboptimal work ability decreasing over time (12%); (2) relatively consistent good work ability (73%) and (3) consistent optimal work ability (15%). The strongest associations with belonging to the suboptimal work ability trajectory were found for employees in work units characterised by high job strain (OR 2.29, 95% CI 1.82 to 2.88), poor team climate (OR 0.74, 95% CI 0.64 to 0.86) and low organisational justice (OR 0.64, 95% CI 0.57 to 0.72) when compared with the most optimal trajectory. The least favourable work ability trajectory was also associated with team reorganisation (OR 1.22, 95% CI 1.04 to 1.44) and a low share of those working from home (OR 0.86, 95% CI 0.78 to 0.94) during the pandemic.

**Conclusion:**

Prepandemic psychosocial risk factors and pandemic-induced changes at work were associated with poor and declining work ability during the COVID-19 pandemic. Employers and occupational health services should better identify and support vulnerable employees to enhance their work participation.

**WHAT IS ALREADY KNOWN ON THIS TOPIC**

- The COVID-19 pandemic affected work and health in various ways. It is probable that during the coronavirus pandemic, many people also experienced changes related to work ability. There are many cross-sectional studies showing an association between work environment and work ability.

**WHAT THIS STUDY ADDS**

- This study adds information on the development of work ability during uncertain times of the COVID-19 pandemic. Moreover, we examine the extent to which work unit characteristics, psychosocial factors and pandemic-related changes contribute to the development of work ability during a crisis.

**HOW THIS STUDY MIGHT AFFECT RESEARCH, PRACTICE OR POLICY**

- Our results indicated that during the pandemic, most employees maintained optimal work ability. However, a smaller group with already suboptimal work ability experienced further decline, partly associated with COVID-related workplace changes. Given the dynamic nature of modern work life, employers and occupational health services should better identify and support these vulnerable employees to enhance their work participation.

## Background

 The COVID-19 pandemic has affected society, work and health in various ways. Societally, the pandemic led to policy interventions such as movement restrictions and recommendations to maintain social distance. In the healthcare system, resources were directed towards the treatment of COVID-19 patients, possibly resulting in disruptions in healthcare services for non-COVID conditions. In the workplace, remote working increased, while in certain sectors, jobs were lost and businesses were shut down. Additionally, the pandemic adversely affected mental health as isolation and uncertainty increased.^1^,^4^ Collectively, pandemic-related changes may have influenced the work ability of many people.

Work ability refers to an individual’s capability to perform work tasks physically, mentally and socially. It encompasses physical health, mental resilience, vocational skills and abilities, and meeting the demands of the work. Factors influencing work ability can be diverse, such as health, skills, working conditions and the work environment. Work ability-related issues may involve healthcare, working conditions, balancing work and family life, and professional development. Organisations strive to maintain and improve their employees’ work ability by providing safe working conditions, healthcare services, vocational support and flexible work arrangements. Preserving work ability is considered an essential part of work well-being and organisational success.^5^

Work unit characteristics and psychosocial resources, including fair decision-making and leadership practices and positive team climate, and risk factors such as job strain, are mutually related. They are also argued to be shared perceptions at the group level, for instance, the work unit.^6 7^ There is a large amount of heterogeneous and primarily cross-sectional studies showing associations between work environment and work ability.^58^,^12^ However, a prospective study^13^ found that the association between working conditions and work ability was negligible. Earlier studies have identified trajectories of work ability,^14^,^17^ but little is known about the development of work ability during the COVID-19 pandemic. Moreover, the extent to which work unit characteristics, psychosocial factors and pandemic-related changes contribute to the development of work ability during a crisis remains unclear.

The current study aimed to examine changes in individual-level work ability from prepandemic to postpandemic periods. Trajectory analysis was used to examine whether there are subgroups with different trajectory paths of work ability over pandemic times. Moreover, we studied whether working in a work unit characterised by good psychosocial resources and low levels of psychosocial risk factors before the pandemic would be associated with maintaining work ability throughout the pandemic and whether the changes to the organisation of work during the pandemic would be associated with the membership in different trajectories.

## Methods

### Study context

The COVID-19 pandemic brought about significant changes in the organisation of work in Finland’s public sector. Employees were transferred to working from home, assigned new tasks or work units were reorganised.^18^ While working from home was associated with an improved psychosocial work environment, other changes, such as transfer into new tasks or a team reorganisation, seemed to relate to somewhat poorer work ability and self-rated health during the pandemic as compared with prepandemic times.^18^

### Research ethics approval

The study follows the Finnish public sector (FPS) protocol in line with the EU and Finnish data legislation. The Ethical Committee of the Helsinki and Uusimaa hospitals approved the FPS study (HUS/1210/2016).

### Study population

**Figure 1:**
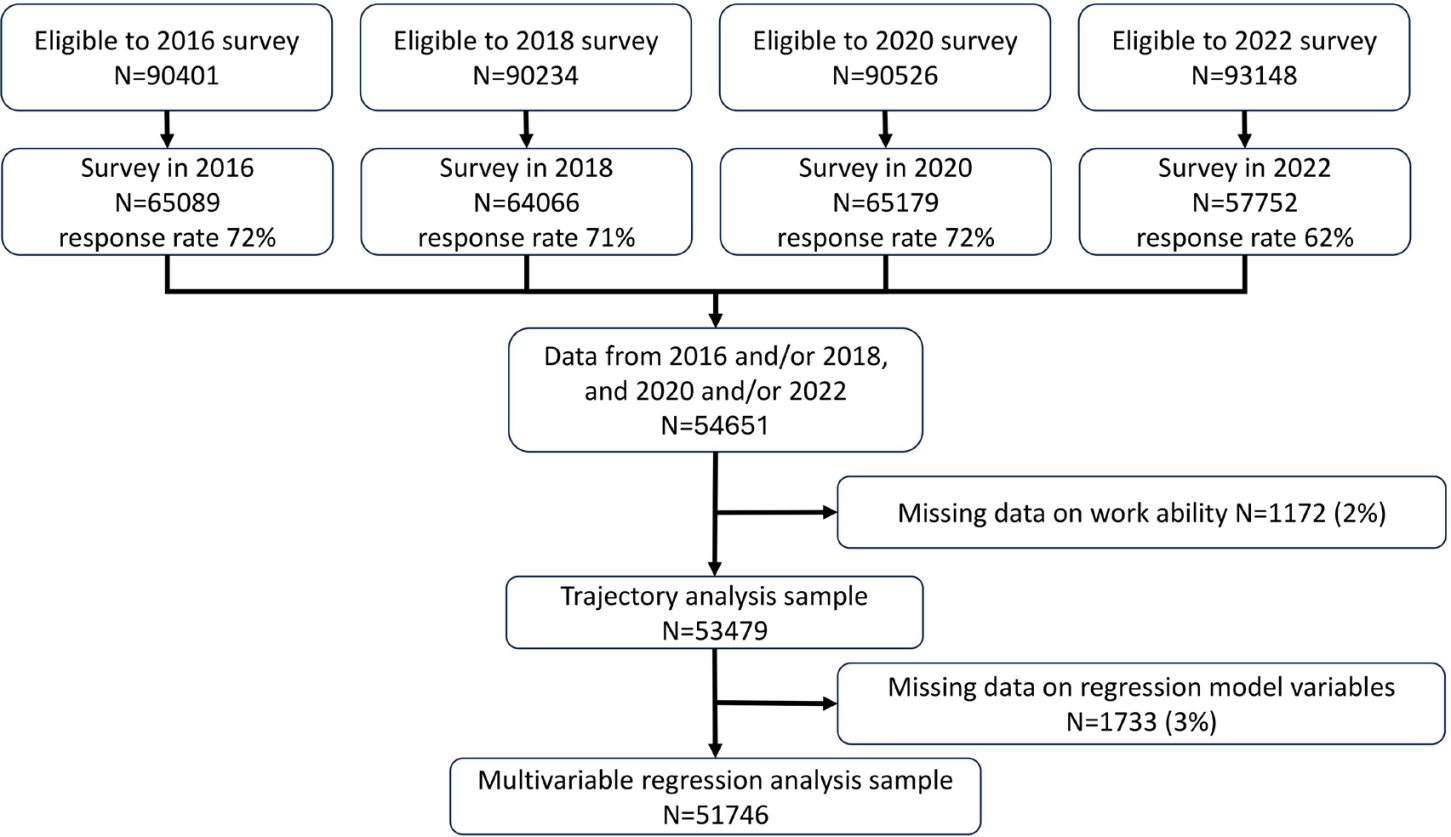
Flow chart illustrating the drawing of the sample for the analyses (Finnish public sector).

The study population was drawn from an ongoing Finnish prospective cohort study, the FPS. FPS is a dynamic cohort of Finnish public sector employees with repeated data collections every 2–4 years initiated from 1997 to 2000 onwards.^18 19^ The eligible population for the present analysis consisted of participants from 11 cities who responded to one or more surveys conducted in 2016 (n=65 089, response rate 72%), 2018 (n=64 066, 71%), 2020 (n=65 179, 72%) and 2022 (n=57 752, 62 %). We included those individuals who responded to the 2016 or 2018 survey (or both), treated as the study baseline and the 2020 or 2022 survey (or both), which served as the follow-up. Additionally, participants had to consent to the linkage of the survey and register data (total 54 651). The number of participants in the work ability trajectory analysis was 53 479 (2.1% missing). In psychosocial work environment trajectories, the number of participants ranged between 54 438 and 54 575 (0.1%–0.4% missing). In the subsequent regression analyses, the number of participants was 51 746 (3.3% missing; figure 1).

### Measures

#### Work ability

We used the work ability score (WAS)^20^ from the work ability index (WAI) questionnaire^21 22^; ‘let’s assume that your work ability at its all-time best would be given 10 points, and 0 points would indicate that you are completely unable to work. How would you score your current work ability?’. WAS has been shown to be a similarly valid but simpler alternative to WAI, which is a subjective estimation of an employee’s work ability in relation to the health status and resources of an employee and the work demands. The WAS has been validated in previous studies.^20^

#### Psychosocial work unit characteristics

*Psychosocial work unit characteristics* at baseline, worktime control, job strain, team climate and organisational justice were retrieved from survey responses and aggregated to work unit level. The aggregate measures of work units were formed as averages of all responses by employees at the lowest level of organisation hierarchy, where there were at least five employees in the work unit.

We measured worktime control using a standard measure. The participants were asked to evaluate on a scale from 1 (very little) to 5 (very much) how much they could influence the following aspects of their working time: length, starting and ending times, breaks and handling of private matters during the workday, scheduling of work shifts, vacations and paid days off and the taking of unpaid leave.^23^

Organisational justice refers to how an organisation treats people fairly, encompassing fairness in outcomes, procedures and interpersonal interactions. Procedural justice as a component of organisational justice involves determining outcomes through unbiased procedures based on accurate information and standard ethics, providing opportunities for input and appeal and ensuring consistency.^24^ Examples of high procedural justice in the workplace include procedures designed to hear the concerns of all employees affected by decisions, collect accurate information necessary for making decisions and allow requests for clarification or additional information about those decisions. Interactional justice pertains to individuals feeling they were treated with dignity and respect and received clear information about procedures and decisions.^25 26^ Organisational justice was measured with a validated scale from 1 (very little) to 5 (very much), combining these two components.^27^

Job strain was measured as a combination of high demands and low job control.^28^ Job demands consisted of five items, which considered time pressures and deadlines, lack of time to do what was expected and work overload. Job control combines two concepts: skill discretion (the opportunities of an individual to develop his or her special abilities within the job, six items) and decision authority (the individual’s abilities to be part of the decision-making process within the organisation, three items). These subscales were combined for the analysis. Responses were given on a five-point scale from 5=strongly agree to 1=strongly disagree. Job strain was defined as high demands (higher than the median score in 2010–2014) and low control (lower than the median score in 2010–2014); all other combinations of job demands and job control were assigned to no strain.^29^

We assessed the work unit cooperation and interaction with the short version of the team climate inventory.^30^ The underlying theory argues that group innovations often result from team activities that are characterised by (1) focusing on clear and realistic objectives to which the team members are committed (vision), (2) interaction between team members in a participative and interpersonally non-threatening climate (participative safety), (3) commitment to high standards of performance and, thus, preparedness for basic questions and appraisal of weaknesses (task orientation) and, finally, (4) enacting support for innovation attempts, including, for example, cooperation to develop and apply new ideas (support innovation). Responses were given on a five-point scale (from 5=strongly agree to 1=strongly disagree).

#### Pandemic-related changes in the organisation of work

*Pandemic-related changes* (working from home, shifting into new tasks or team reorganisation) and baseline work unit characteristics were derived from individual survey responses aggregated to the work unit level. During the pandemic in 2020, we asked the participants whether the COVID-19 pandemic had caused the following changes in their work: (1) the employee was transferred partially or totally to working from home; (2) the employee was transferred to new work tasks within the same occupational sector or to another occupational sector and/or (3) their work unit/team was reorganised into a smaller or larger unit. Each participant could have faced none, one or more of these changes.

#### Covariates

*Prepandemic work unit characteristics* of age, sex and socioeconomic status (SES) distributions were retrieved from employers’ registers in 2016. SES based on occupational titles was classified according to the 2001 International Standard Classification of Occupations codes into high (upper-grade non-manual workers, including managers, administrators and specialists) and low (lower-grade non-manual workers, including office workers, clerks, customer service and sales workers, nurses and construction, manufacturing, and transportation workers). Other characteristics included job tenure, part-time versus full-time work and temporary versus permanent job contracts.

Individual-level confounders, body mass index (BMI), alcohol use, smoking and physical inactivity from the baseline survey were adjusted for in the analyses.^18^ We calculated BMI from self-reported weight and height. Excessive alcohol consumption was determined based on weekly alcohol units.^31^ Smoking status was based on a question ‘Do you smoke?’, and it was dichotomised into ‘smokers’ (daily or occasional smokers) and ‘non-smokers’ (never or former smokers, reference group).^32^ Physical activity was enquired by asking respondents how much they exercised in general and transformed into weekly metabolic equivalent task (MET) hours.^33^ Physical inactivity was categorised as ‘inactive’ if weekly MET hours were less than 14, and as ‘active’ (the reference group) if more than that.

### Statistical analysis

We used trajectory analysis to identify developmental trajectories of work ability. Work ability was treated as a continuous variable. We evaluated and compared model fit with Bayesian Information Criteria (BIC), Akaike Information Criteria (AIC), log-likelihood, average posterior probability, smallest group, logged Bayes factor and relative entropy.^34 35^ We also set a predefined cut point for the smallest group, ≥5%, as in a previous study.^36^

We used multinomial regression analysis to determine the extent to which baseline work unit characteristics, work unit psychosocial factors and pandemic-induced changes in work organisation were associated with the work ability trajectories. The aggregate measures of work units were formed as averages of all responses by employees at the lowest level of the organisation hierarchy, where there were at least five employees in the work unit. McFadden’s pseudo-R^2^ was calculated to estimate the total percentage explained by the independent variables included in the models.

We performed trajectory analyses with SAS V.9.4 (proc traj). For multinomial regression analyses, we used R (V.4.3.0) with nnet-package (V.7.3-18).

## Results

### Trajectories in the context of the COVID-19 pandemic

**Figure 2:**
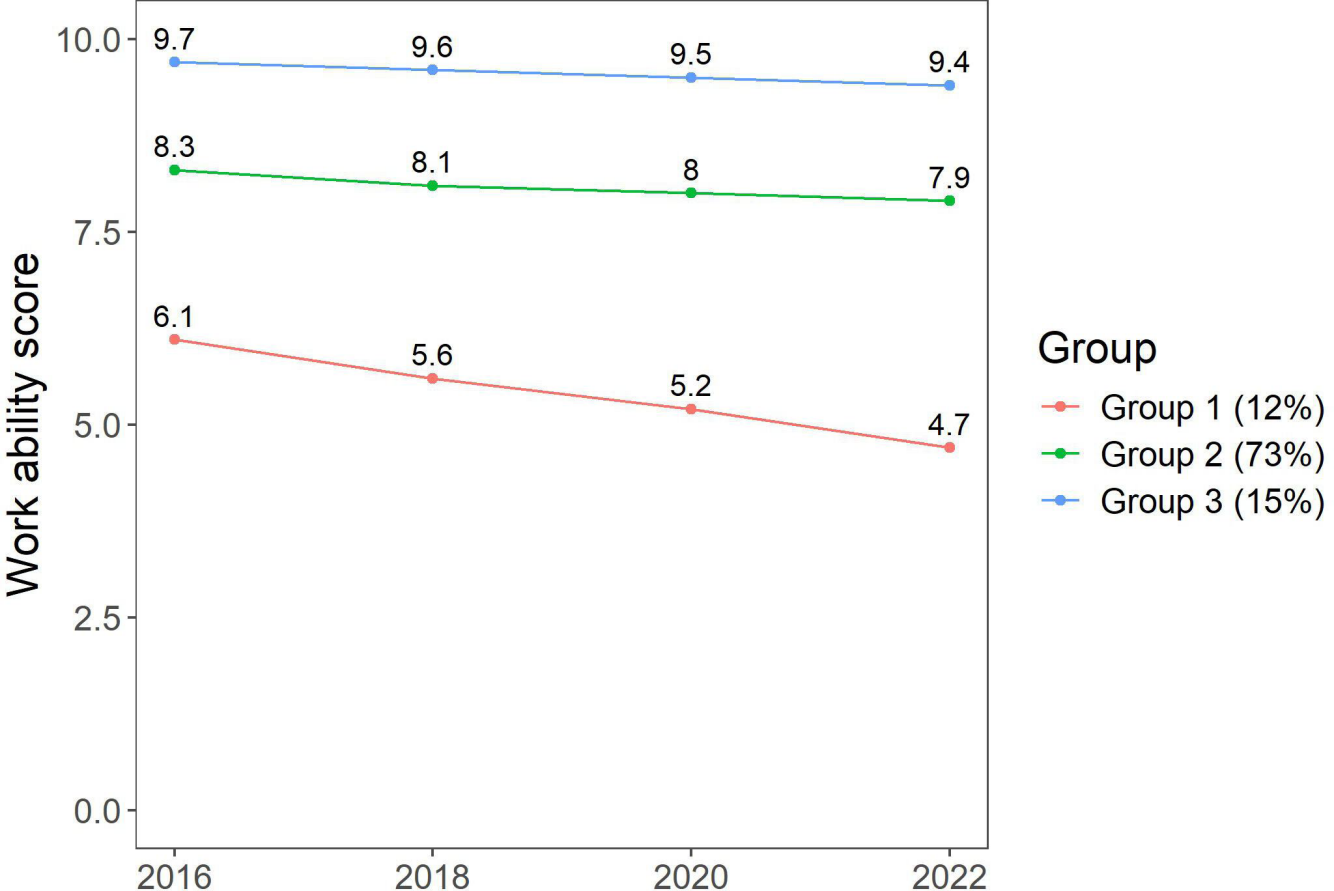
Three trajectories of work ability score. Trajectory 1: suboptimal work ability decreasing over time (12%); Trajectory 2: relatively consistent good work ability (73%) and Trajectory 3: consistent optimal work ability (15%).

The goodness of fit estimates for 1–5 trajectory solutions for work ability from before the pandemic to after the pandemic are shown in table 1. The model fit indices were acceptable even for models with 4–5 work ability trajectories. Based on the predefined model fit criteria, we chose a three-trajectory model. With acceptable BIC, AIC and log-likelihood values, the prevalence of the smallest group was 12% in this trajectory solution, and posterior probabilities were above 0.80. While BIC and AIC values decreased as the number of trajectories increased, the decrease was less pronounced after the three-trajectory solution. In addition, the Bayes factor comparing the change in BIC between models was significantly smaller in a model with four trajectories than in a model with three trajectories, suggesting that the relative improvement in model fit from three to four trajectories was modest. The trajectories are illustrated in figure 2:

1. Suboptimal work ability decreasing over time (12%).
2. Relatively consistent good work ability (73%).
3. Consistent optimal work ability (15%).

**Table 1:** Model fit statistics for work ability score trajectory models (n=53 479)

No. of trajectories	1	2	1	2	3	4	5
No. of parameters	3	6	2	4	**6**	8	10
Polynomial order	2	22*	1	11	**111**	1111	11 111
BIC	−325187.8	−314456.5	325 182.4	−307800.7	**−300392.8**	−297402.3	−295581.0
Akaike Information Criteria	−325170.0	−314420.9	−325169.1	−307774.0	**−300352.8**	−297349.0	−295514.3
Log-likelihood	−325166.0	−314412.9	−325166.1	−307768.0	**−300343.8**	−297337.0	−295499.3
Average posterior probabilities	1	–	1	0.89/0.97	**0.91/0.93/0.85**	0.91/0.90/0.82/0.87	0.89/0.79/0.75/0.89/0.87
Smallest group	100%	50%	100%	17%	**12%**	4%	3%
2×∆BIC (Bayes factor)		–		34 763.4	14 815.8	5981	3642.6
Relative entropy		–		0.84	**0.81**	0.80	0.79

The selected/chosen model is highlighted in bold.

* The trajectory model ‘2 2’ had false convergence. Average posterior probabilities, Bayes factor, or relative entropy are not calculated.

BICBayesian Information Criteria

Compared with the two more optimal work ability trajectories, employees belonging to the suboptimal and decreasing work ability trajectory were more often men, older, in lower occupational positions, had longer job tenure and had more often a part-time job but less often a temporary job contract. They also had a higher BMI, and they were more often physically inactive, used more alcohol and smoked more often (table 2).

**Table 2:** Individual characteristics by work ability trajectory membership

	Consistent optimal work ability (n=7302)	Relatively consistent good work ability (n=40 052)	Suboptimal work ability decreasing over time (n=6125)
Men, %	23.9	19.9	24.5
Women, %	76.1	80.1	75.5
Mean age (SD)	41.7 (9.9)	44.6 (9.8)	47.4 (9.2)
High SES, %	47.7	46.0	35.7
Middle SES, %	26.1	26.2	27.2
Low SES, %	26.2	27.8	37.1
Job tenure, years	9.4	12.1	14.4
Full-time job, %	96.5	96.1	92.8
Part-time job, %	3.5	3.9	7.2
Permanent employment, %	80.0	85.2	90.1
Temporary employment, %	20.0	14.8	9.9
Mean body mass index (SD)	24.6 (3.9)	26.0 (4.7)	27.8 (5.6)
Non-smoking, %	90.6	87.5	82.0
Smoking, %	9.4	12.5	18.0
Mean alcohol use* (SD)	39.9 (71.2)	46.2 (75.7)	56.4 (112.2)
Mean physical activity† (SD)	7.7 (5.7)	5.5 (4.5)	4.3 (4.0)

* In grams of pure alcohol in a week.

† MET etabolic equivalent task hours in a day.

SESsocioeconomic status

We also analysed the trajectory solutions for the psychosocial work environment: organisational justice (procedural and relational justice), team climate, worktime control and job strain. The model fit indices and figures of best trajectory solutions are shown in online supplemental tables and figures W1–5. Overall, we found that the trajectories of the psychosocial work environment did not change during the follow-up; that is, we found practically no increasing or decreasing trajectories. Thus, psychosocial factors were derived from prepandemic time, aggregated to work unit level, and used as predictors of the work ability trajectories.

### Work unit factors associated with work ability trajectories

Work unit factors before COVID-19 were interrelated: work units with better team climate also had higher organisational justice (Pearson r=0.65). Work units with high job strain had poorer team climate (r=−0.30) and lower organisational justice (r=−0.25). Considering pandemic-related changes, working from home was more common in work units with higher SES (r=0.63) and lower job strain (r=−0.39) (online supplemental table W6).

The unadjusted and multivariable associations of baseline work unit characteristics, work unit psychosocial factors and pandemic-induced changes with work ability trajectory membership are shown in table 3. The most optimal trajectory, ‘consistent optimal work ability’ was used as the reference trajectory.

**Table 3:** Baseline work unit psychosocial factors and pandemic-induced changes associated with work ability trajectories

		Unadjusted		Multivariable	
		OR	95% CI	OR	95% CI
**Relatively consistent good work ability, 73%,n=40 052**					
Prepandemic work unit psychosocial factors	Work time control	0.94	0.90 to 0.98	0.94	0.90 to 0.99
	Job strain	1.80	1.57 to 2.07	1.45	1.22 to 1.73
	Team climate	0.75	0.70 to 0.81	1.00	0.90 to 1.11
	Organisational justice	0.72	0.67 to 0.76	0.78	0.71 to 0.85
Pandemic-induced changes at work	Working from home	0.77	0.71 to 0.84	0.99	0.92 to 1.05
	New tasks	1.03	0.83 to 1.27	0.96	0.87 to 1.05
	Reorganisation	1.41	0.98 to 2.03	1.19	1.05 to 1.34
**Suboptimal work ability decreasing over time, 12%,n=6125**					
Prepandemic work unit psychosocial factors	Work time control	0.92	0.87 to 0.97	0.92	0.86 to 0.99
	Job strain	4.52	3.78 to 5.41	2.29	1.82 to 2.88
	Team climate	0.39	0.35 to 0.43	0.74	0.64 to 0.86
	Organisational justice	0.45	0.41 to 0.49	0.64	0.57 to 0.72
Pandemic-induced changes at work	Working from home	0.50	0.44 to 0.56	0.86	0.78 to 0.94
	New tasks	1.14	0.86 to 1.52	1.01	0.89 to 1.14
	Reorganisation	1.56	0.97 to 2.53	1.22	1.04 to 1.44

Reference=consistent optimal work ability, 125%, n=7302. Adjusted for work unit sex, age, and socioeconomic status distributions, work unit mean job tenure, share of part-time and temporary job contracts, and individual-level smoking, alcohol use, body mass index, and physical activity. ratio, interval.

After adjusting for individual-level health behaviours, BMI and baseline work unit characteristics, trajectory membership of ‘relatively good consistent work ability’ was associated with work units with lower organisational justice (OR 0.78, 95% CI 0.71 to 0.85), lower worktime control (OR 0.94, 95% CI 0.90 to 0.99) and higher job strain (OR 1.45, 95% CI 1.22 to 1.73) as compared with the most optimal trajectory. During the pandemic, members of this trajectory were more probably exposed to team reorganisation (OR 1.19, 95% CI 1.05 to 1.34) as compared with those in the most optimal work ability trajectory (table 3).

Trajectory membership of ‘suboptimal work ability decreasing over time, 12%, n=6125’ was associated with even poorer team climate (OR 0.74, 95% CI 0.64 to 0.86), lower organisational justice (OR 0.64, 95% CI 0.57 to 0.72), lower worktime control (OR 0.92, 95% CI 0.86 to 0.99) and higher job strain (OR 2.29, 95% CI 1.82 to 2.88) as compared with the most optimal trajectory. During the pandemic, members of this trajectory were more probably in on-site work (OR 0.86, 95% CI 0.78 to 0.94) and were exposed to team reorganisation (OR 1.22, 95% CI 1.04 to 1.44) as compared with employees in the most optimal trajectory (table 3).

The McFadden pseudo-R^2^ of the multivariable logistic modelling was 0.047, indicating that we were only able to explain 5% of the total variance in work ability trajectory profiles. To examine whether the low overall coefficient of determination was due to work unit-level predictor variables, we performed a sensitivity analysis with individual-level predictor variables presented in online supplemental table W7. Individual-level variables did not have a marked effect on the pseudo-R^2^, which increased only to 8%.

## Discussion

This prospective cohort study followed 54 651 public sector employees over 6 years, from prepandemic to postpandemic period, to identify the developmental trajectories of work ability. Additionally, we explored whether work unit characteristics and psychosocial work environment of the work units before the pandemic and changes in work organisation during the pandemic were associated with membership in the trajectories.

Three work ability trajectories emerged: (1) suboptimal work ability decreasing over time, (2) relatively consistent good work ability and (3) consistent optimal work ability. This study contributes to identifying factors that predispose to adverse development of work ability in the context of the COVID-19 pandemic. In line with earlier studies on work ability trajectories, while most work ability trajectories remained rather stable over the follow-up period, we did also identify an adverse pattern.^14 15 17 37^

The time span from before the COVID-19 pandemic to the time after the pandemic offers a unique viewpoint on work unit characteristics contributing to pandemic resilience in workplaces, that is, to maintaining work ability in times of crisis. We found that employees in units characterised by high job strain, poor team climate and low organisational justice were more likely to belong to the least favourable work ability trajectory. They were less likely to have worked from home during the pandemic and more likely to have been exposed to a reorganisation of teams at the workplace.

However, it is also noteworthy that while we did find some work unit characteristics associated with adverse development of work ability during the pandemic, our modelling was only able to explain a small amount of the total variance in the trajectory profiles. This may be, in part, explained by our observation that, overall, psychosocial work environment factors did not markedly change during the follow-up.

### Strengths and limitations

A strength of this study is the large sample of public sector workers and a prospective study setting with a relatively long follow-up time catching a specific period of the COVID-19 pandemic. Our data included a rich set of both individual and psychosocial work environment factors assessed with well-established scales. We used trajectory modelling, which is a ﬂexible statistical tool that represents the development of indicators over time. In addition, to reduce the effect on individual response style and common source bias, we aggregated the work environment factors to the work unit level. Thus, the measurement of the psychosocial work environment was not based on the same source as used for developmental trajectories. While participants evaluated their work ability repeatedly over time, the psychosocial variables used in the regression modelling combined the responses to the psychosocial work environment from all respondents within that work unit. With these work unit-level measures of the psychosocial work environment, we examined individual-level work ability. WAS has been found to be a strong predictor of future long-term sickness absence and disability pension.^20 38^

As our sample consisted of public sector employees, the results may not be generalisable to other sectors. The time span in our study is particularly interesting due to COVID-19; however, it is unclear whether our results can be generalised to other types of crises of modern working life. Also, pandemic-induced changes at work were not mutually exclusive.

We were able to observe the development of psychosocial resource factors related to work ability through the pandemic. These are modifiable factors that could affect pandemic resilience within organisations, although our results suggest the total variance in work ability explained by these factors is relatively modest. Thus, it is likely that numerous other factors have also influenced work ability trajectories during the pandemic. Moreover, in the most adverse work ability trajectory, the level of work ability was suboptimal even before the pandemic. Thus, we cannot make causal conclusions about the role of the pandemic in the worsening of work ability. However, the pandemic context makes this study unique.

Selection bias due to non-response and missing values is possible. However, response rates were relatively high (ranging from 62% to 72%) in each round, and missing values accounted for no more than 2% of the trajectory analyses. Such analyses are generally considered robust to missing values.^39^ Our regression analyses required complete information from all exposures and covariates, leading to slightly more exclusions, primarily due to missing BMI data. However, the reduction in the sample was modest, at 3%, making it an unlikely source of significant bias in our findings. Our results indicated that during the pandemic, most employees maintained optimal work ability despite the changes in the psychosocial work environment. However, a smaller group with already suboptimal work ability experienced a further decline, partly due to COVID-related workplace changes. Given the dynamic nature of modern work life, employers and occupational health services should better identify and support these vulnerable employees to enhance their work participation.

## supplementary material

10.1136/oemed-2024-109475 online supplemental file 1

## Data Availability

Data are available upon reasonable request.
