# Supplementary material for: Trajectories of work ability and associated work unit characteristics from pre-COVID to post-COVID pandemic period
Source: Occup Environ Med. 2024 Nov 13;81(11):e109475. doi: 10.1136/oemed-2024-109475 (PMC11672023; doi:10.1136/oemed-2024-109475)
Supplement: online supplemental file 1 [file oemed-81-11-s001.pdf]

Web appendices

Contents

Trajectory solutions for psychosocial work environment characteristics in the context of COVID-19 pandemic 2

Table W1. Model fit statistics for procedural justice trajectory models (N=53479). 2

Figure W1. Four trajectories of procedural justice. 3

Table W2. Model fit statistics for relational justice trajectory models (N=54438). 4

Figure W2. Three trajectories of relational justice. 5

Table W3. Model fit statistics for team climate trajectory models(N=54460). 6

Figure W3. Three trajectories of team climate. 7

Table W4. Model fit statistics for worktime control trajectory models (N=54575). 8

Figure W4. Four trajectories of worktime control. 9

Table W5. Model fit statistics for job strain (=binary) trajectory models (N=54536). 10

Figure W5. Two trajectories of job strain. 11

Table W6. Correlation matrix. 12

Table W7. Baseline individual-level psychosocial factors and pandemic-induced changes associated with work ability trajectories. 13

Trajectory solutions for psychosocial work environment characteristics in the context of COVID-19 pandemic

Table W1. Model fit statistics for **procedural justice** trajectory models (N=53479).

The model with four trajectories was chosen, as in the model with five trajectories, the smallest group was only 2%.

N of trajectories	N of parameters	Polynomial order	BIC	AIC	Log-likelihood	Average posterior probabilities	Smallest group	2 × ΔBIC (Bayes factor)	Relative entropy
1	3	2	-235436.5	-235418.7	-235414.7	1	100%		
2	6	2 2 *	-224153.6	-224118.1	-224110.1	-	50%	-	-
1	2	1	-235431.0	-235417.7	-235414.7	1	100%		
2	4	1 1	-219331.7	-219305.1	-219299.1	0.89/0.88	48%	32198.6	0.64
3	6	1 1 1	-213912.4	-213872.4	-213863.4	0.85/0.86/0.85	17%	10838.6	0.69
**4**	**8**	**1 1 1 1**	**-212504.7**	**-212451.4**	**-212439.4**	**0.83/0.79/0.80/0.81**	**8%**	**2815.4**	**0.66**
5	10	1 1 1 1 1	-212069.0	-212002.4	-211987.4	0.77/0.80/0.77/0.80/0.76	2%	871.4	0.67

*Note. The trajectory model had false convergence. Average posterior probabilities, Bayes factor, or relative entropy are not calculated.

Group 1: Consistently low procedural justice (10%)
Group 2: Consistently rather high procedural justice, slightly increasing during the pandemic (43%)
Group 3: Consistently moderate procedural justice (39%)
Group 4: Consistently high procedural justice, slightly increasing during the pandemic (8%)

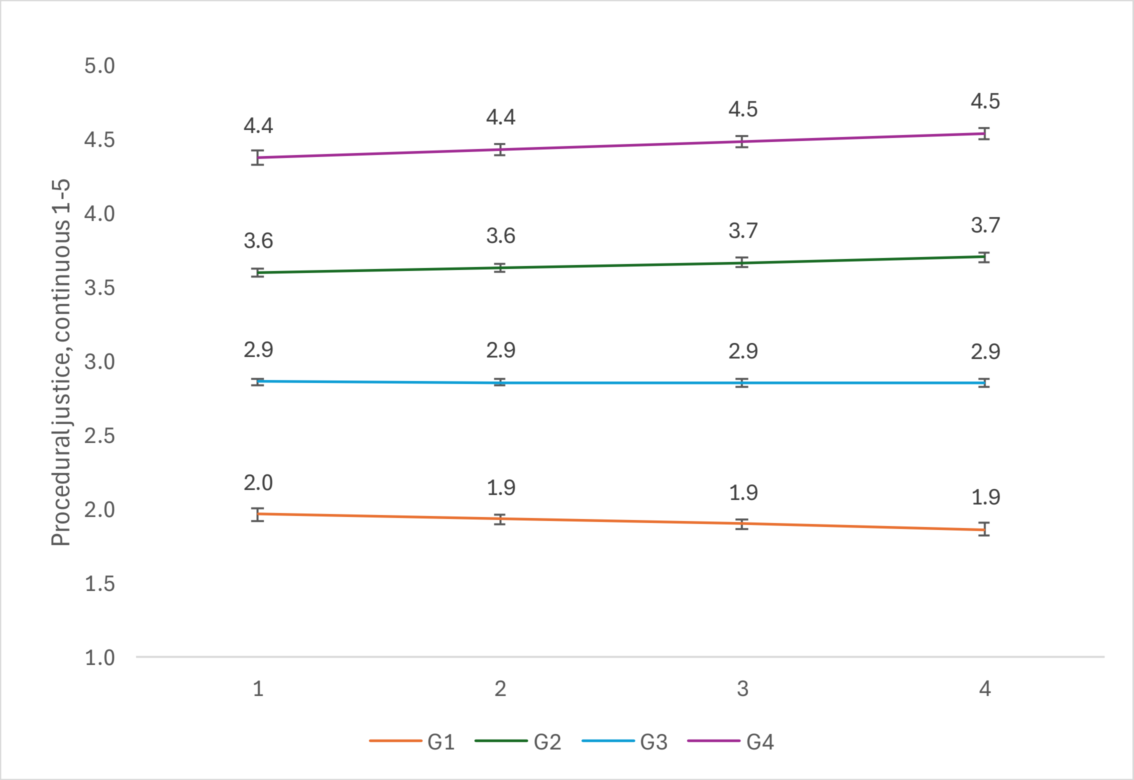

Figure W1. Four trajectories of procedural justice.

Table W2. Model fit statistics for **relational justice** trajectory models (N=54438).

The model with three trajectories was chosen, as in the model with four to five trajectories, the smallest group was only 3%.

N of trajectories	N of estimates	Polynomial order	BIC	AIC	Log-likelihood	Average posterior probabilities	Smallest group	2 × ΔBIC (Bayes factor)	Relative entropy
1	3	2	-243309.4	-243291.6	-243287.6	1	100%		
2	6	2 2 *	-230985.0	-230949.4	-230941.4	0.85/0.92	30%	24648.8	0.66
1	2	1	-243304.0	-243290.6	-243287.6	1	100%		
2	4	1 1	-230974.1	-230947.4	-230941.4	0.85/0.92	30%	24659.8	0.66
**3**	**6**	**1 1 1**	**-227659.5**	**-227619.4**	**-227610.4**	**0.85/0.81/0.83**	**9%**	**6629.2**	**0.63**
4	8	1 1 1 1	-226890.0	-226836.6	-226824.6	0.83/0.77/0.78/0.76	3%	1539	0.63
5	10	1 1 1 1 1	-225585.6	-225518.8	-225503.8	0.81/0.78/0.77/0.69/0.70	3%	2608.8	0.64

* Note. The 95% confidence intervals of the 2 trajectories ranged from 1 to 5.

Group 1: Consistently low relational justice (9%)
Group 2: Consistently rather high relational justice (52%)
Group 3: Consistently high relational justice (39%)

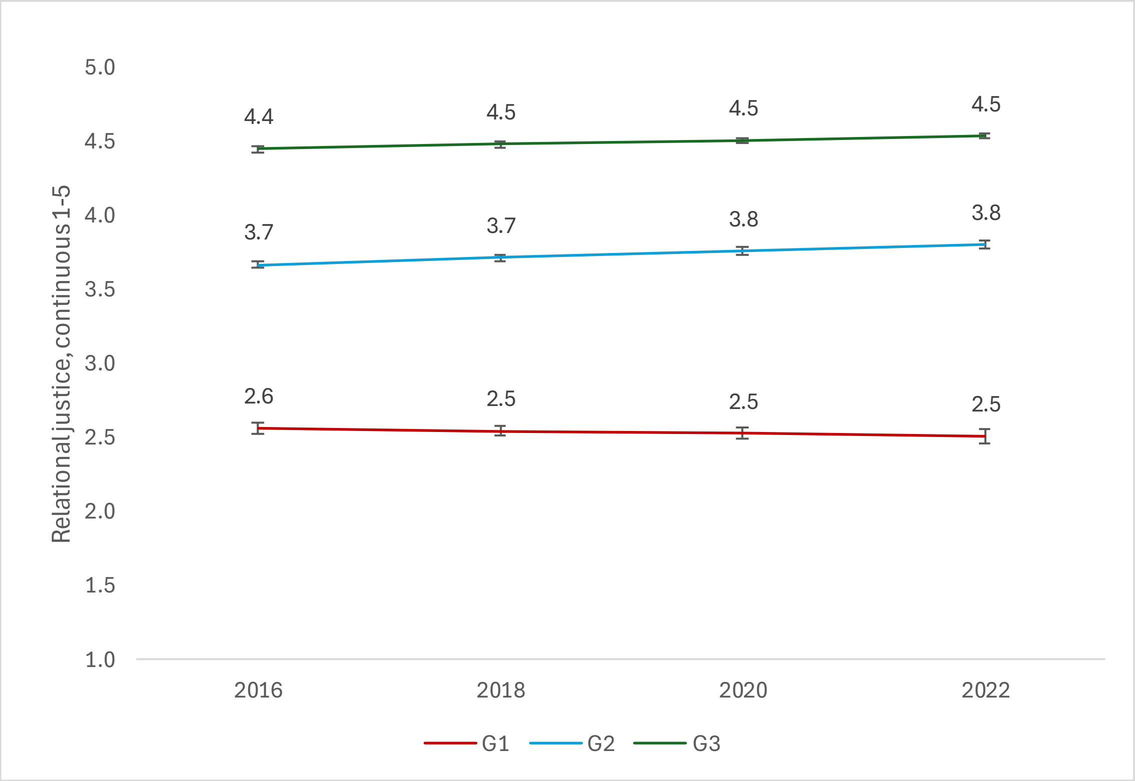

Figure W2. Three trajectories of relational justice.

Table W3. Model fit statistics for **team climate** trajectory models(N=54460).

The model with three trajectories was chosen, as in the model with four trajectories, the smallest group was only 3%.

N of trajectories	N of estimates	Polynomial order*	BIC	AIC	Log-likelihood	Average posterior probabilities	Smallest group	2 × ΔBIC (Bayes factor)	Relative entropy
1	3	2	-177298.1	-177280.3	-177276.3	1	54460 (100%)		
2	6	2 2	-158546.9	-158511.2	-158503.2	0.88/0.93	34%	37502.4	0.70
1	2	1	-177292.7	-177292.7	-177276.3	1	54460 (100%)		
2	4	1 1	-158535.9	-158509.2	-158503.2	0.88/0.93	34%	37513.6	0.70
**3**	**6**	**1 1 1**	**-151882.5**	**-151842.4**	**-151833.4**	**0.88/0.85/0.86**	**13%**	**13306.8**	**0.70**
4	8	1 1 1 1	-149623.6	-149570.2	-149558.2	0.87/0.83/0.81/0.82	3%	4517.8	0.70

* Note. The 95% confidence intervals of the 2 trajectories ranged from 1 to 5.

Group 1: Consistently poor team climate (13%)
Group 2: Consistently high team climate (33%)
Group 3: Consistently moderate team climate (54%)

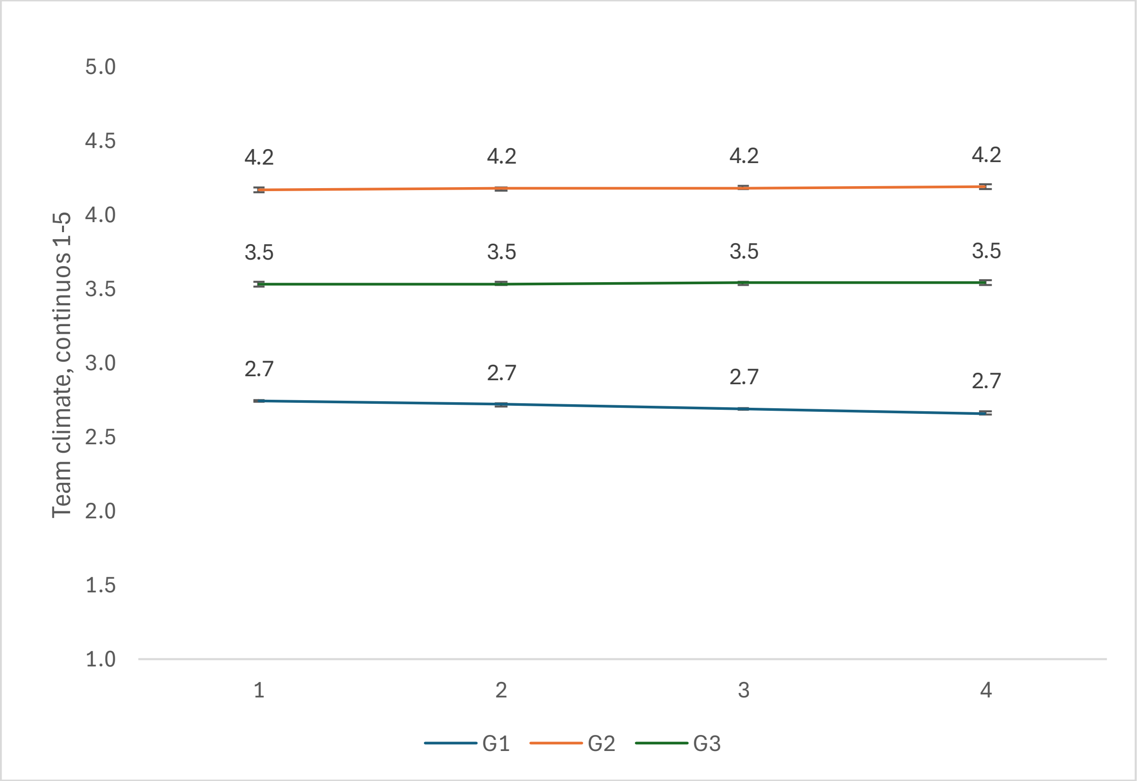

Figure W3. Three trajectories of team climate.

Table W4. Model fit statistics for **worktime control** trajectory models (N=54575).

The model with four trajectories was chosen, as in the model with five trajectories, the smallest group was only 5%.

N of trajectories	N of estimates	Polynomial order*	BIC	AIC	Log-likelihood	Average posterior probabilities	Smallest group	2 × ΔBIC (Bayes factor)	Relative entropy
1	3	2	-237470.8	-237453.0	-237449.0	1	100%		
2	6	2 2 *	-199128.7	-199093.1	-199085.1	0.95/0.94	43%	76684.2	0.82
1	2	1	-237465.5	-237452.1	-237449.1	1	100%		
2	4	1 1	-199117.9	-199091.2	-199085.2	0.95/0.94	43%	76695.2	0.82
3	6	1 1 1	-185844.1	-185804.0	-185795.0	0.92/0.89/0.92	22%	26547.6	0.80
**4**	**8**	**1 1 1 1**	**-180976.7**	**-180923.3**	**-180911.3**	**0.89/0.85/0.86/0.90**	**11%**	**9734.8**	**0.77**
5	10	1 1 1 1 1	-178993.2	-178926.4	-178911.4	0.87/0.81/0.86/0.85/0.81	5%	3967	0.75

* Note. The 95% confidence intervals of the 2 trajectories ranged from 1 to 5.

Group 1: Consistently low worktime control (24%)
Group 2: Consistently rather high worktime control, slightly increasing during the pandemic (37%)
Group 3: Consistently moderate worktime control (28%)
Group 4: Consistently high worktime control, slightly increasing during the pandemic (11%)

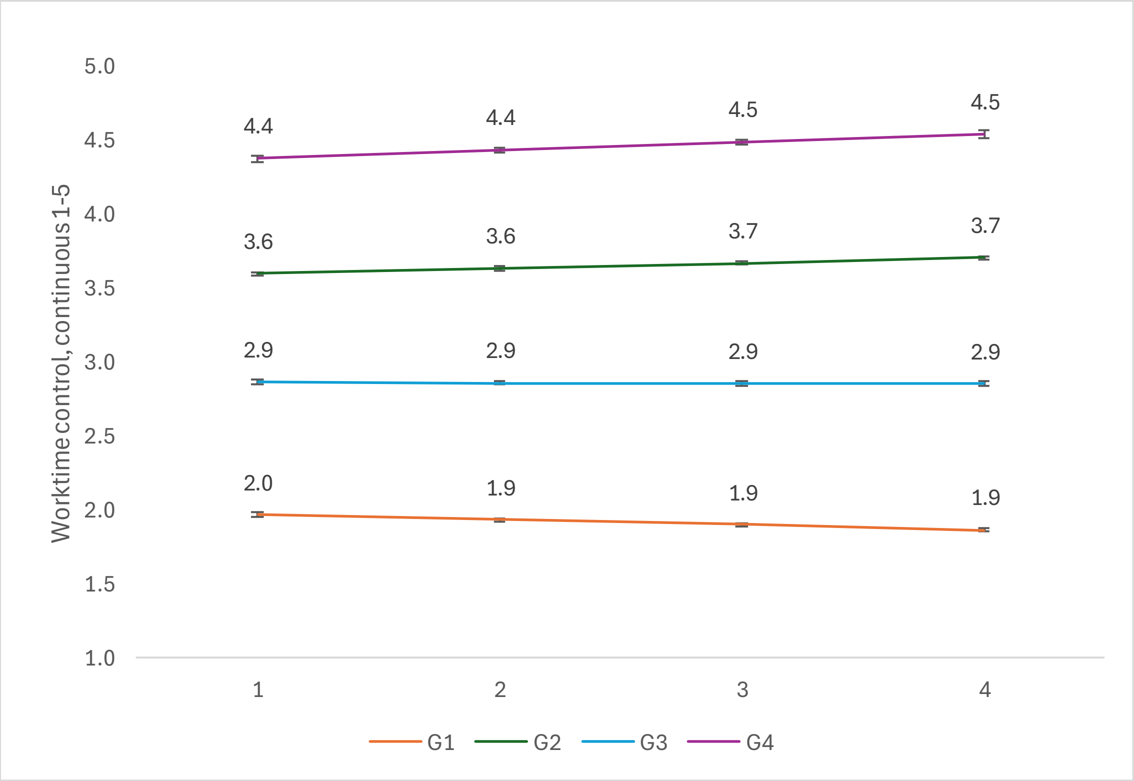

Figure W4. Four trajectories of worktime control.

Table W5. Model fit statistics for **job strain** (=binary) trajectory models (N=54536).

The model with two linear trajectories was chosen, as the model with three trajectories had singular convergence and model with a linear and a quadratic order had poorer model fit indices.

N of trajectories	N of estimates	Polynomial order*	BIC	AIC	Log-likelihood	Average posterior probabilities	Smallest group	2 × ΔBIC (Bayes factor)	Relative entropy
1	3	2	-94117.2	-94103.9	-94100.9	1	100%		
2	6	2 2 *	-84307.5	-84276.4	-84269.4	0.93/0.85	27%	19619.4	0.68
1	2	1	-94111.7	-94102.8	-94100.8	1	100%		
**2**	**4**	**1 1**	**-84296.5**	**-84274.3**	**-84269.3**	**0.93/0.85**	**27%**	**19630.4**	**0.68**
3	6	1 1 1 **	-84164.02	-84128.4	-84120.4	0.89/0.74/0.61	5%	264.96	
2		2 1	-84302.1	-84275.4	-84269.4	0.93/0.85	27%	-11.2***	0.68

* Note. The 95% confidence intervals of the 2 trajectories ranged from 1 to 5.

** Note. ERROR: Singular convergence.

*** Comparison to model with linear order for both trajectories (polynomial order ‘1 1’)

Group 1: Low job strain, very slightly increasing during and after the pandemic (73%)
Group 2: High job strain, increasing in time irrespective of the pandemic (27%)

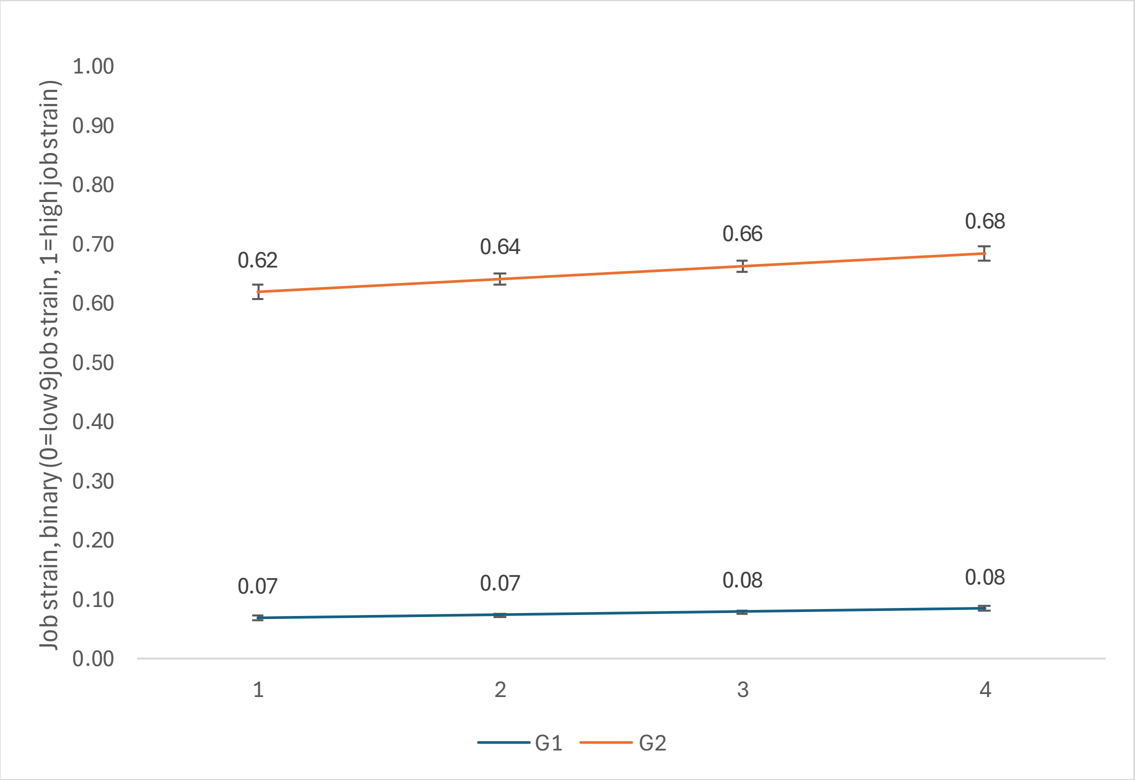

Figure W5. Two trajectories of job strain.

Table W6. Correlation matrix.

1-18 are individual-level variables; 1a-14a are aggregated, work unit level variables. Red marks high negative correlations, green marks high positive correlations.

	INDIVIDUAL-LEVEL VARIABLES																		WORK UNIT LEVEL VARIABLES													
	1 Sex	2 Age	3 High SES	4 Low SES	5 Job tenure	6 Part-time	7 Temporary	8 Worktime control	9 Job strain	10 Team climate	11 Organizational justice	12 Transfer into working from home	13 Transfer into other tasks	14 Team reorganization	15 Smoking	16 Alcohol use	17 BMI	18 MET hours	1a % Women	2a Mean age	3a % High SES	4a % Low SES	5a Mean job tenure	6a % Part-time	7a % Temporary	8a Mean worktime control	9a Mean Job strain	10a Mean team climate	11a Mean organizational justice	12a Mean working from home	13a Mean transfer into other tasks	14a Mean team reorganization
2	-0.01	-																														
3	0.01	0.03	-																													
4	-0.01	-0.03	-0.57	-																												
5	-0.02	0.60	-0.01	0.02	-																											
6	-0.06	-0.03	-0.04	0.02	-0.03	-																										
7	-0.01	-0.21	0.06	-0.06	-0.24	0.05	-																									
8	0.07	-0.02	-0.06	-0.15	-0.03	0.02	-0.01	-																								
9	-0.07	0.01	-0.18	0.17	0.01	0.02	-0.04	-0.18	-																							
10	-0.05	-0.04	0.10	-0.03	-0.01	-0.01	0.05	0.10	-0.23	-																						
11	0.00	0.03	-0.09	0.01	0.04	0.00	-0.07	-0.17	0.22	-0.59	-																					
12	0.07	0.01	0.43	-0.47	-0.03	-0.05	0.04	0.14	-0.14	-0.01	-0.01	-																				
13	-0.07	-0.03	-0.14	0.04	-0.02	0.05	0.00	-0.02	0.04	0.00	0.02	-0.11	-																			
14	-0.02	0.02	-0.04	0.04	0.02	0.01	-0.01	-0.05	0.01	0.01	0.01	-0.08	0.11	-																		
15	0.07	0.00	-0.22	0.16	0.00	0.00	0.02	0.05	0.04	-0.07	0.06	-0.11	0.01	0.00	-																	
16	0.26	0.06	0.03	-0.03	0.06	-0.04	0.00	0.05	-0.04	-0.04	0.03	0.04	-0.02	-0.01	0.14	-																
17	0.09	0.12	-0.12	0.09	0.09	-0.02	-0.05	0.05	0.03	-0.05	0.05	-0.05	-0.01	0.00	0.09	0.07	-															
18	0.11	-0.16	0.07	-0.05	-0.11	-0.02	0.05	-0.03	-0.04	0.06	-0.03	0.04	-0.01	0.00	-0.08	0.00	-0.24	-														
1a	0.64	0.01	-0.01	-0.03	0.01	-0.06	-0.04	0.07	-0.09	-0.11	0.06	0.12	-0.10	-0.04	0.06	0.19	0.07	0.07	-													
2a	0.03	0.36	0.06	-0.07	0.23	-0.01	-0.08	0.09	-0.02	-0.08	0.03	0.10	-0.01	-0.01	-0.01	0.04	0.05	-0.08	0.05	-												
3a	0.00	0.03	0.76	-0.45	-0.01	-0.01	0.06	-0.11	-0.17	0.09	-0.08	0.48	-0.14	-0.04	-0.20	0.02	-0.12	0.05	-0.01	0.08	-											
4a	-0.03	-0.04	-0.46	0.74	0.03	-0.01	-0.06	-0.14	0.16	-0.02	0.01	-0.55	0.03	0.06	0.13	-0.04	0.09	-0.04	-0.05	-0.10	-0.61	-										
5a	0.04	0.21	0.00	0.03	0.41	-0.03	-0.11	0.00	-0.02	-0.02	0.00	-0.02	-0.01	0.02	-0.02	0.04	0.04	-0.04	0.06	0.61	0.00	0.04	-									
6a	-0.11	0.01	-0.02	-0.02	-0.02	0.33	0.02	-0.01	0.04	0.00	0.00	-0.07	0.10	0.03	-0.02	-0.04	-0.03	-0.02	-0.18	0.04	-0.03	-0.02	-0.04	-								
7a	-0.06	-0.08	0.10	-0.12	-0.11	0.03	0.40	-0.02	-0.03	0.05	-0.03	0.09	-0.01	-0.01	-0.01	-0.02	-0.04	0.04	-0.09	-0.24	0.13	-0.16	-0.30	0.08	-							
8a	0.07	0.05	-0.11	-0.15	0.00	-0.01	-0.02	0.70	-0.08	-0.05	-0.02	0.16	-0.01	-0.06	0.08	0.04	0.04	-0.04	0.11	0.12	-0.14	-0.21	0.00	-0.02	-0.02	-						
9a	-0.14	-0.01	-0.28	0.28	-0.01	0.03	-0.03	-0.12	0.44	-0.15	0.13	-0.29	0.06	0.03	0.07	-0.07	0.04	-0.05	-0.22	-0.05	-0.37	0.37	-0.06	0.10	-0.08	-0.18	-					
10a	-0.14	-0.05	0.13	-0.03	0.00	0.00	0.05	-0.06	-0.13	0.51	-0.32	-0.02	0.02	0.02	-0.08	-0.06	-0.07	0.05	-0.22	-0.16	0.16	-0.04	-0.03	0.00	0.10	-0.09	-0.30	-				
11a	0.08	0.02	-0.12	0.01	-0.01	0.00	-0.03	-0.02	0.11	-0.33	0.49	-0.02	0.02	0.00	0.06	0.04	0.04	-0.01	0.13	0.06	-0.15	0.01	-0.01	0.01	-0.07	-0.03	0.25	-0.65	-			
12a	0.10	0.04	0.48	-0.51	-0.02	-0.04	0.05	0.11	-0.17	0.01	-0.03	0.72	-0.12	-0.08	-0.12	0.05	-0.07	0.05	0.16	0.10	0.63	-0.69	-0.02	-0.12	0.09	0.17	-0.39	0.02	-0.06	-		
13a	-0.11	-0.01	-0.18	0.03	0.00	0.06	-0.01	-0.01	0.04	0.02	0.01	-0.15	0.54	0.10	0.00	-0.04	-0.02	-0.01	-0.17	-0.03	-0.23	0.04	-0.01	0.16	-0.03	-0.01	0.09	0.04	0.02	-0.17	-	
14a	-0.06	0.00	-0.06	0.09	0.03	0.02	-0.01	-0.10	0.02	0.03	0.00	-0.14	0.13	0.40	0.00	-0.01	0.00	-0.01	-0.09	-0.03	-0.08	0.12	0.05	0.05	-0.03	-0.14	0.06	0.06	-0.01	-0.16	0.25	-

Table W7. Baseline individual-level psychosocial factors and pandemic-induced changes associated with work ability trajectories.

Reference=Consistent optimal work ability, 12%, n=4903. Adjusted for sex, age, socioeconomic status, part-time vs. full-time work, the length of work contract, temporary vs. permanent job contract, smoking, alcohol use, body mass index, and physical activity. OR=odds ratio, CI=confidence interval.

		Relatively good consistent work ability, 73% n=30759				Declining work ability, 15% n=4841			
		Bivariate		Multivariate		Bivariate		Multivariate	
		OR	95% CI	OR	95% CI	OR	95% CI	OR	95% CI
	Work time control	0.84	0.81, 0.87	0.88	0.85, 0.92	0.67	0.64, 0.70	0.75	0.72, 0.80
	Job strain	1.75	1.60, 1.92	1.39	1.25, 1.53	3.48	3.14, 3.87	1.98	1.76, 2.23
	Team climate	0.58	0.55, 0.61	0.69	0.65, 0.74	0.31	0.29, 0.33	0.50	0.46, 0.55
	Organizational justice	0.65	0.63, 0.68	0.81	0.77, 0.85	0.4	0.38, 0.42	0.65	0.60, 0.69
	Working from home	0.89	0.84, 0.95	0.96	0.89, 1.03	0.64	0.59, 0.70	0.90	0.81, 0.99
	New tasks	1.02	0.92, 1.14	0.97	0.86, 1.09	1.14	0.99, 1.31	1.06	0.90, 1.23
	Reorganization	1.30	1.12, 1.50	1.31	1.11, 1.53	1.38	1.15, 1.66	1.26	1.03, 1.54
